# Modulation of lymphatic transport in the central nervous system

**DOI:** 10.7150/thno.66026

**Published:** 2022-01-01

**Authors:** Wenzhong Li, Dawei Chen, Nan Liu, Yongxin Luan, Shoujun Zhu, Haifeng Wang

**Affiliations:** 1 Department of Neurosurgery, The first hospital of Jilin University, Changchun, 130021, P.R. China; 2 Department of Anesthesiology, The first hospital of Jilin University, Changchun, 130021, P.R. China; 3 Joint Laboratory of Opto-Functional Theranostics in Medicine and Chemistry, The First Hospital of Jilin University, Changchun, 130021, P.R. China; 4 State Key Laboratory of Supramolecular Structure and Materials, College of Chemistry, Jilin University, Changchun 130012, P.R. China

**Keywords:** central nervous system, glymphatic system, lymphatic vessels, regulation, sleep

## Abstract

Over the past decade, repeated studies demonstrated that the vertebrate brain had a specialized lymphatic transport pathway, which overturned the traditional concept of central nervous system (CNS) immune privilege. Despite the lack of lymphatic vessels, the glymphatic system and the meningeal lymphatic vessels provide a unique pathway for solutes transport and metabolites clearance in the brain. Sleep, circadian rhythm, arterial pulsation, and other physiological factors modulate this specialized lymphatic drainage pathway. It has also changed significantly under pathological conditions. These modulatory mechanisms may arise critical targets for the therapeutic of CNS disorders. This review highlights the latest research progress on the modulation of lymphatic transport in the CNS under physiological and pathological conditions. Furthermore, we examined the possible upstream and downstream relation networks between these regulatory mechanisms.

## Introduction

The lymphatic transport system in vertebrates maintains body fluid homeostasis, immune surveillance, and lipid reabsorption in the peripheral organs; it also plays an essential role in the pathological process underlying inflammation, cardiovascular disease, tumor metastasis, and in the onset of other diseases 1. The human brain consumes about 20% of total energy 2. It, therefore, needs a more effective fluid transport system to ensure a stable microenvironment supporting its active metabolic status. Studies have recently identified and characterized a specialized lymphatic transport system in the central nervous system (CNS) 3-6. It is responsible for immune monitoring, solute transport, metabolites clearance, and other functions similar to those of the peripheral lymphoid system; also involved in the pathological process of CNS disorders, such as tumors, head trauma, stroke, and degenerative diseases 7.

**Figure 1:**
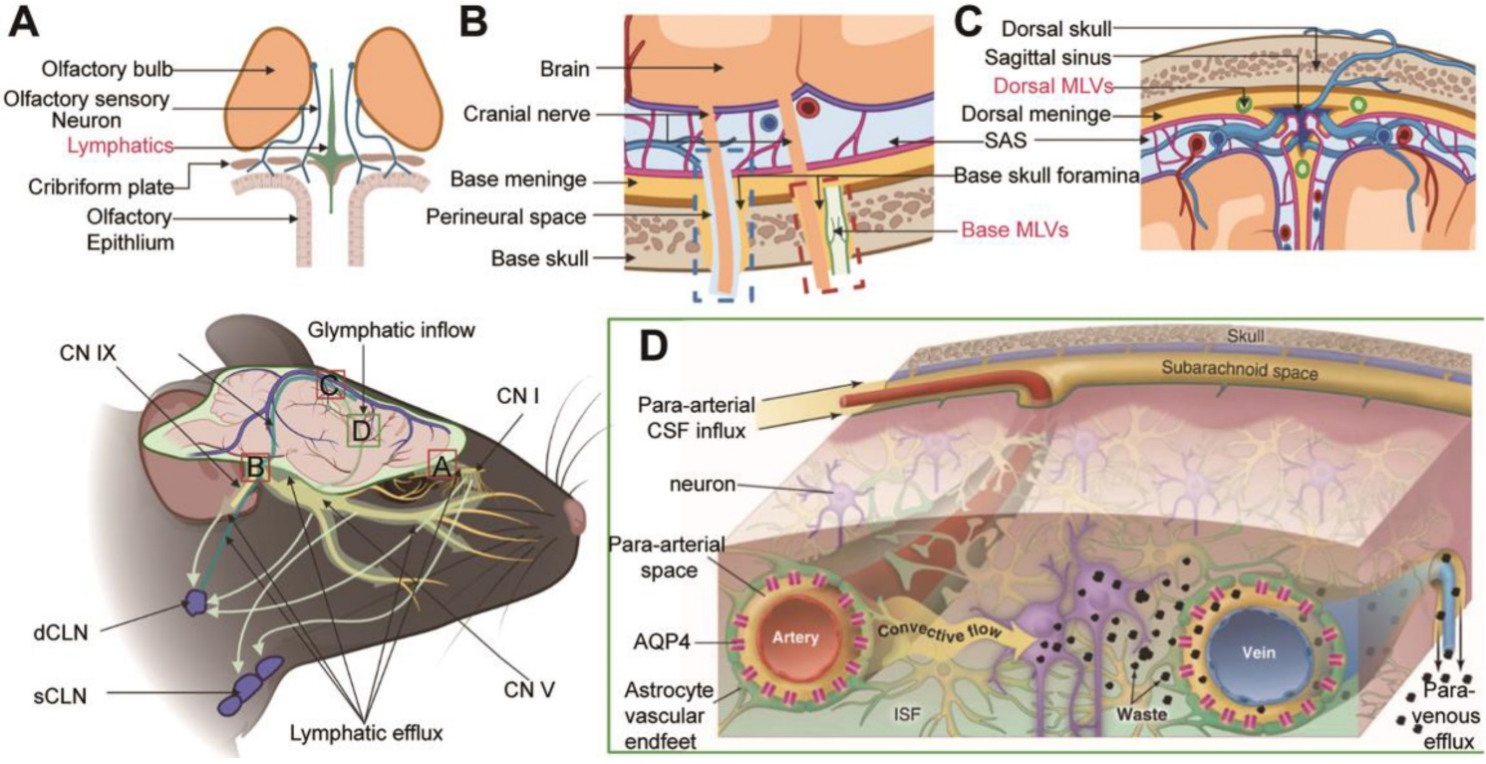
**Schematic diagram of lymphatic transport in the CNS.** (A) Lymphatic vessels near the cribriform plate. (B) Two uncertain pathways for CSF drainage at the base skull: the peripheral pathways (blue dashed box) and the base MLVS that overlap with the nerve anatomically (red dashed box). (C) Dorsal MLVs near sagittal sinus. (D) Overview of the lymphatic transpor**t** of CSF and ISF through the glymphatic system. Note: CN I, olfactory nerve; CN V, trigeminal nerve; CN IX, glossopharyngeal nerve; CN X, vagus nerve. Adapted with permission from Ref. 124 for A, copyright 2019 Nature Publishing Group; Ref. 125 for D, copyright 2013 American Association for the Advancement of Science. Created with BioRender.com.

For a long time, researchers have suggested that cerebrospinal fluid (CSF) may be the "sink" through which brain metabolites is cleared 8. Since CSF is the carrier solution for metabolites clearance in the brain, these studies are similar to those investigating CSF dynamics in the CNS. As traditionally thought to buffer the brain and spinal cord, CSF is now accepted as the lymph of the CNS. The lymphatic transport system in the CNS has been studied primarily in the context of two discoveries: the glymphatic system (GS) and meningeal lymphatic vessels (MLVs) (Figure 1). The GS, named after the 'glial' and 'lymphatic' waste clearance systems, depicts a fluid pathway through which CSF flows in and out of the brain 3: CSF flows into the brain parenchyma (it is also referred to as glymphatic inflow in this review) through the artery paravascular space (aPVS), then exchanges solutes with interstitial fluid (ISF) in the interstitial system (ISS), and finally flows out of the brain via the venous paravascular space (vPVS) 3, 9 (Figure 1D). Although the brain lacks lymphatic vessels, studies have identified unique lymphatic structures in the basal and dorsal meninges 5, 10-12 (Figure 1B-C). These MLVs are also engaged in the outflow of CSF, metabolites, and immune cells from the brain 5, 10-12. Multiple pathways may contribute to CSF drainage into the extracranial lymphatic vessels (in this review, we generally name them lymphatic efflux). Recent studies showed that CSF mainly flowed out of the cranial cavity via the MLVs 6, 10. Moreover, the GS and MLVs have interrelated anatomical structures and functions; some researchers name them the glymphatic-lymphatic fluid transport system 9, 13.

Numerous studies have focused on this specialized lymphatic transport system's physiological modulation and pathological changes 14-18. This review highlights the current research progress on the modulation of lymphatic transport in the CNS, focusing on two main routes: glymphatic inflow and lymphatic efflux (Figure 1). In particular, the main topic of these studies reviewed herein is the glymphatic inflow, a critical pathway that transports lymph into the brain to perform. Importantly, no single physiological and pathological factor can explain this complex regulatory process in isolation. We summarize the internal connection between the upstream and downstream physiological modulation and the potential relationship between physiological modulation and pathological changes in the GS.

## Modulation of the glymphatic inflow

### Natural sleep

**Figure 2:**
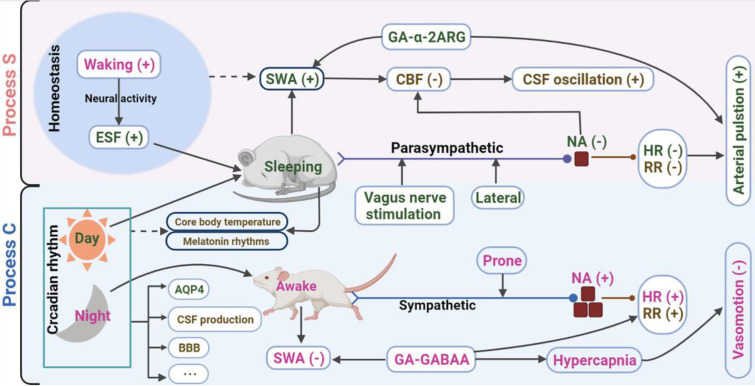
**The possible upstream and downstream relation networks between physiological modulatory factors.** Note: The factors in dark green font represent promoted effects on the glymphatic influx, according to the current study; pink, inhibition; brown, unclear. Two dark blue boxes display the markers of processes S and C. "+", increased; "-", decreased. GA-α-2ARG, general anesthesia with α-2 adrenergic receptor agonist; GA-GABA_A_, general anesthesia with a positive allosteric modulator of GABA_A_; HR, heart rate; NA, norepinephrine; RR, respiratory rate. Created with BioRender.com.

Sleep is a naturally occurring physiological state of decreasing arousal. During non-rapid eye movement (NREM) sleep, more CSF flows into ISS, and the brain's metabolites clearance is significantly more efficient than that in awake 14. Low sleep quality is a risk factor for CNS diseases, such as Alzheimer's disease (AD), migraine, and dementia, which may be related to the brain's less clearance of solutes/metabolites 18-21. For the complex regulatory mechanism of sleep, various regulation models have been proposed in the past few decades. The two-process model of sleep regulation, which assumes the interaction between the homeostatic recovery process (process S) and circadian pacemaker process (process C), is widely recognized in sleep research 22. Since the glymphatic inflow is closely related to sleep, some factors regulating sleep may be potential factors modulating glymphatic inflow, and these factors may also have internal relations (Figure 2).

#### Slow-wave activity and general anesthesia

**Figure 3:**
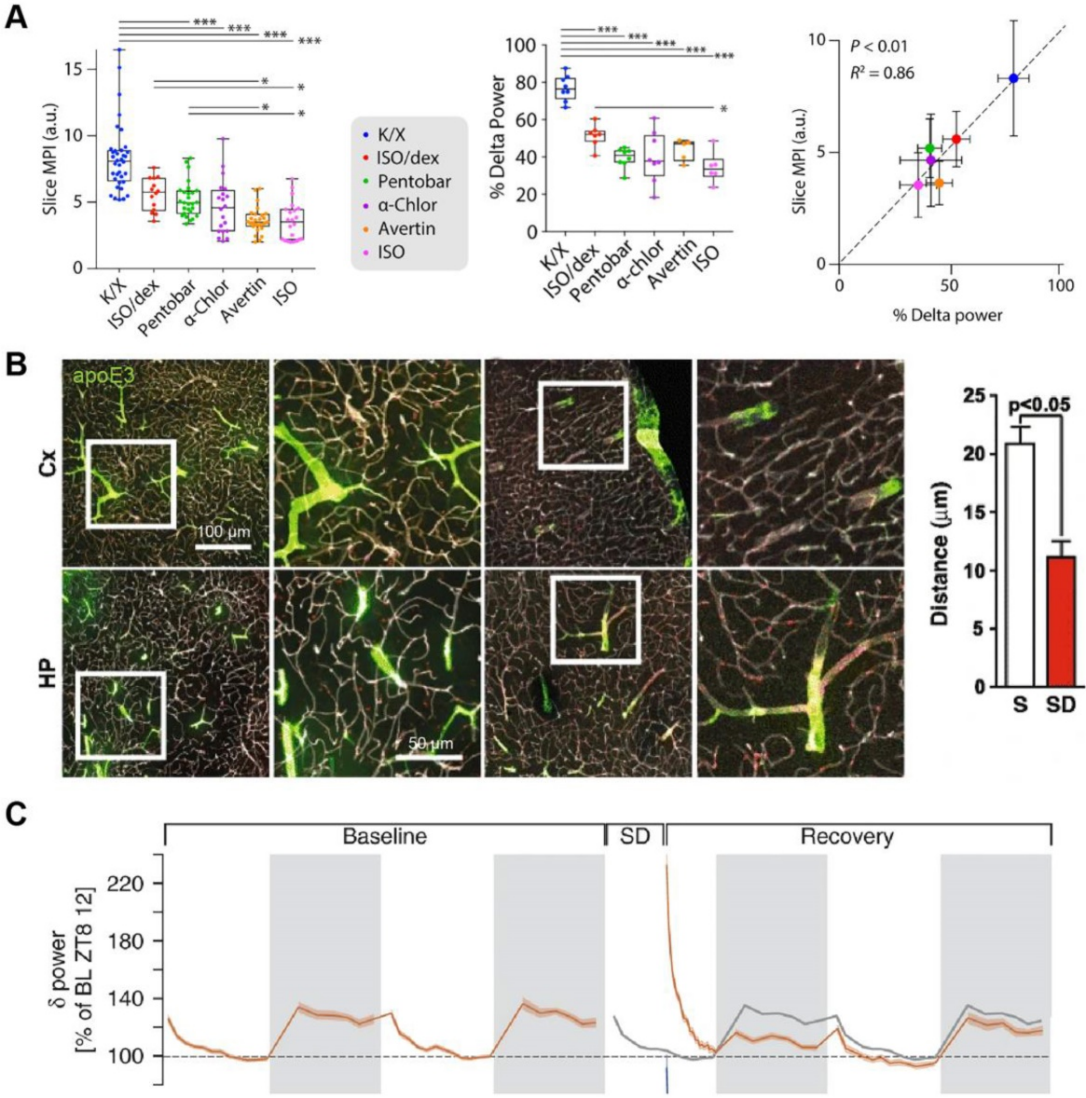
**Relationship between the glymphatic inflow and SWA.** (A) SWA correlates with the various efficiencies of different anesthesia on the glymphatic influx. (B) The inflow distance of fluorescently-tagged apoE3 decreased in sleep-deprived mice. (C) The SWA of the ensuing sleep after sleep deprivation increased. Adapted with permission from Ref. 26 for A), copyright 2019 American Association for the Advancement of Science; Ref. 31 for B), copyright 2016 BioMed Central; and Ref. 35 for C), copyright 2020 Nature Publishing Group.

NREM sleep electroencephalography (EEG) slow-wave activity (SWA, 0.5-4Hz; a combination of slow oscillation and delta oscillations) represents the principal marker of the process S during sleep 22. A pivotal study demonstrates that oscillations of CSF flow are interlinked with SWA in human sleep 23. The addition of SWA accompanied by pulsatile CSF flow during sleep may be the internal mechanism leading to higher metabolite clearance 23. During NREM sleep, the power density of SWA increases, and the glymphatic inflow increases consistently 14. General anesthesia (GA) is a non-physiological and reversible drug-induced state, which is different from the neurophysiological mechanism of natural sleep 24. Different general anaesthetics or sedatives have distinct inhibitory effects on the GS. Some anesthetics show positive functions similar to those observed during spontaneous sleep, while others are significant adverse, and these diverse effects are associated with SWA 15, 25, 26 (Figure 3A).

Still, there are some unclear and even confusing issues. As a marker of neurons' spontaneous and rhythmic electrical activities, EEG has been a leading tool to study brain function in health and disease. However, much less is known about its content, given the complex relationship between EEG features and microcircuit structure 27. Existing terms typically refer to the frequency band that the rhythm occupies rather than its mechanism 28. SWA during sleep is synchronized with the relatively short resting period of cortical neurons. At the same time, anesthesia-induced SWA is related to the significant enhancement of inhibiting postsynaptic currents (IPSCs) in the cortical loop, which is not synchronized with the long-term resting period of cortical neurons 24. As a parameter to model process S in the two-process model, some literature has indicated that SWA regulates independently of sleep, which may be an epiphenomenon of sleep 29, 30. Future insightful studies on the upstream neurophysiology of the origin of SWA will enable us to understand the differential effects of sleep and anesthesia on the GS.

Besides, some studies found that the glymphatic inflow decreased following sleep deprivation 31, 32 (Figure 3B). It is a confusing conclusion because the change in SWA during re-sleep after sleep deprivation is complicated. NREM sleep is affected by the prior sleep-wake history, and the SWA increases in the ensuing sleep stage after sleep deprivation, while they decrease gradually with prolonged sleep time 33-35 (Figure 3C). The increased SWA after sleep deprivation contradicts the inhibition of glymphatic inflow observed by these results. Of course, it is more likely a comprehensive process because these studies also discovered that the decreased glymphatic inflow after sleep deprivation was associated with the decline of paravascular polarization of the aquaporin-4 (AQP4) 31, 32. Compared to humans, mice exhibit fragmented sleep patterns, characterized by unabiding sleep bouts, frequent awakenings, and a short (only 10-20 minutes) ultradian NREM / REM cycle duration 36. Therefore, if the SWA modulates the GS, monitoring the EEG in an accurately defined sleep phase is necessary to reevaluate the relationship between sleep deprivation, SWA, and the GS.

Furthermore, current researches on glymphatic transport rely on tracers of larger molecular solutes to simulate CSF transport, which may underestimate the flow of CSF water into brain parenchyma. The classical tracer study measured AQP4-dependent paracellular flow of the liquid exchange of ISS confirmed that tracers inflow were related to molecular solute size 3. The use of H_2_^17^O to capture both paracellular flow and diffusive transcellular exchange of water showed a faster and more active glymphatic transport 37. The increased extracellular volume fraction is another possible reason for the increased CSF tracers inflow during sleep or ketamine/xylazine (K/X) cocktail anesthesia 14. As the H_2_^17^O tracer freely travels through the intracellular and extracellular pathways, extracellular volume fraction will also have a minor impact on the water transport of CSF 37. Both paracellular flow and diffusive transcellular exchange of water are AQP4-dependent 38, 39.

#### Autonomic nervous system

During NREM sleep, the inhibition of the adrenergic system increases the area of the ISS; consequently, it reduces the fluid resistance and increases the solute transport efficiency 14. The discharge of local neurons will spread to vast or local brain regions, which control multiple downstream targets 28. Investigating the downstream effects after recording the electrophysiological activities may help us understand the physiological regulation of the glymphatic inflow.

The sympathetic tone is reduced during natural sleep, whereas the parasympathetic tone is increased 40. Sleep initiation comes from the activation of γ-aminobutyric acid (GABA) and galanin receptors and the projective inhibition of synapses in the ascending reticular activated system neurons 36, 41. Xylazine and dexmedetomidine inhibit the release of norepinephrine from the locus coeruleus by binding to the α^-2^ adrenergic receptors; this effect is consistent with the inhibition of norepinephrine release during natural sleep 42. Both xylazine and dexmedetomidine show higher CSF tracers inflow efficiency than other anaesthetics 26. Pentobarbital, α-chloralose, tribromoethanol, and isoflurane can enhance GABA-induced chloride influx and IPSCs by activating the GABA-A receptors 43-45, and these anaesthetics significantly inhibit glymphatic inflow 15, 26. CSF tracers inflow decreases considerably in the case of acute hypertension induced by epinephrine 46. Vagal nerve stimulation effectively cures migraines and AD, enhancing the glymphatic inflow 47. Mammals prefer the lateral position during sleep. The glymphatic influx was more effective in the right lateral decubitus than in the prone position 48. Moreover, the prone position increases sympathetic tone, while the vagal tone is increased in the right lateral position, which may be the reason for the increased glymphatic inflow 48, 49.

Summarizing these results, we speculate that the autonomic nervous system (ANS) has a wide range of modulatory effects on the glymphatic inflow. The ANS also regulates physiological parameters such as respiration, heart rate, and vascular pulsation. Some studies examined the impact of physiological parameters on GS, which were reviewed later in this paper.

#### Endogenous sleep factors

Endogenous sleep factors (ESF) such as adenosine, nitric oxide, and prostaglandin D2 have significant efficiency in regulating NREM sleep homeostasis (process S) 41. Previous studies investigated the effects of these sleep factors on cerebral blood flow (CBF) 50, 51. Owing to the coupling relationship between CBF and glymphatic influx 23, these ESF may also be potential molecules regulating the glymphatic inflow. In rats, low dose alcohol intake can promote glymphatic influx and metabolites clearance by inducing nitric oxide production and vasodilation 52. Cocaine reduces adenosine transporter activity 53, and mice administered with cocaine have impaired glymphatic pathways 54. Indeed, the potential value of these endogenous molecules has been noticed in the CNS diseases such as Alzheimer's disease, brain injury, stroke 55-57. Future studies need to generate more evidence to reveal the mechanism and contribution of ESF toward regulating the clearance systems in the brain. Modulating NREM sleep through the intervention of these ESF may be an effective strategy to enhance brain clearance function.

#### Circadian rhythm

The circadian rhythm controlled by the suprachiasmatic nucleus of the hypothalamus plays the role of a pacemaker in the two-process model of sleep regulation 22, 58. The GS is further modulated by circadian rhythm. The glymphatic inflow reaches the peak during possible daytime sleeping of mice 17. The circadian rhythm of AQP4 perivascular polarization could be an internal mechanism of glymphatic influx affected by circadian rhythm 17. Circadian rhythm governs CSF production, blood-brain barrier (BBB) permeability, and plasma norepinephrine concentration 59-61. We need further ascertain whether these factors, modulated by the circadian rhythm, contribute to the circadian rhythm of the glymphatic inflow. In addition, it is also worth studying whether the two markers of process C, core body temperature and melatonin rhythms, can regulate the glymphatic inflow.

### Cerebral artery pulsation and physiological parameters

Many physiological factors, including intracranial pressure (ICP), blood pressure, heart rate, and respiratory, regulate the fluid transport of the cerebrovascular system and PVS 62. Since PVS provides a path for the glymphatic inflow, these factors may also be potential driving forces for the glymphatic inflow (Figure 2).

#### Arterial pulsation

**Figure 4:**
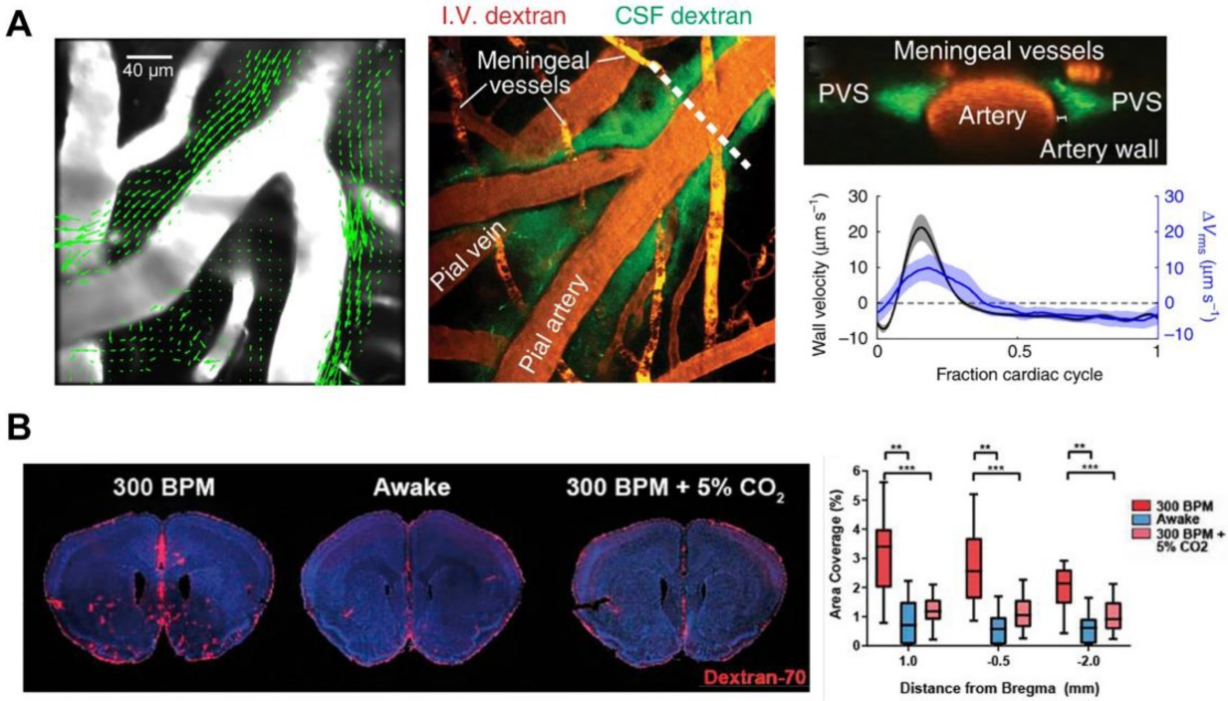
**The driving force of brain waste clearance.** (A) aPVS has a large cross-sectional area, and the particles flow convectively in the direction of blood. The peak in-wall velocity and the delay time are consistent with the peak and delay of the spatial root means square velocity curve. (B) Hypercapnic ventilation significantly reduces CSF tracer influx in K/X-anesthetized mice. Adapted with permission from Ref. 46 for A), copyright 2018 Nature Publishing Group; Ref. 74 for B), copyright 2020 SAGE Publications Inc.

It is still controversial about the fluid transport mode in the ISS: convection or diffusion. Some modeling studies showed that arterial pulsation alone could not provide sufficient driving force to account for convective transport due to the narrow aPVS 63, 64. Nonetheless, *in vivo* two-photon imaging have demonstrated that aPVS was a flat, double tubular eccentric structure, 1.4 times (whereas fixation reduced this ratio to 0.14) the cross-sectional area of the adjacent artery with low fluid resistance 46 (Figure 4A). The transport of intrathecal contrast agents through the brain is faster than expected from diffusion alone in humans 65. Exploiting an isotopically enriched MRI tracer, H_2_^17^O, a recent study revealed that the glymphatic transport was dramatically faster and more extensive than previously thought 37, further supporting the convective movement of paracellular CSF water. These studies advantageously support that arterial pulsation is the primary driving force of ISF convection. The flow of CSF in PVS is a whole pulsatile flow, which follows the same direction as that of the blood flow and has a similar frequency with the cardiac cycle 46 (Figure 4A). In humans, phase-contrast magnetic resonance imaging studies have shown that enhanced intracranial arterial pulsation promoted the influx of contrast molecules into aPVS 66. When the internal carotid artery was ligated to inhibit the pulse of the cortical perforating artery, the glymphatic inflow was impaired; in contrast, dobutamine-enhanced pulsation of the perforating artery was positively correlated with the increased glymphatic influx 67. Hypertension is also a risk factor for neurodegenerative diseases such as Alzheimer's and Parkinson's 68, 69. Epinephrine-induced acute hypertension leads to arteriosclerosis, decreased vascular compliance, increased CSF reflux, and reduced CSF transport efficiency in aPVS 46.

#### Respiration rate and heart rate

The flow of CSF in the ventricular system and subarachnoid spaces (SAS) is mainly regulated by respiration, while the contribution of cardiac pulsations on this process is low 70. To date, there is no direct evidence to prove that respiration modulates the glymphatic inflow, although respiration is the main factor influencing CSF drainage to the peripheral lymph nodes 62. Given that rapid CSF efflux inhibits the glymphatic influx, further experiments are needed to identify the effect of respiration on the glymphatic inflow 71. Mice treated with different anaesthetics showed that increased glymphatic influx was associated with decreased heart rate 26. This study also indicated that respiration and blood pressure were not associated with glymphatic influx 26. However, it should be noted that GA significantly reduces the respiratory dynamics, leading to hypercapnia, which can inhibit the outflow to the lymph nodes and inflow to the brain of CSF 72-74 (Figure 4B). Considering the contribution of the glymphatic influx to the metabolite clearance and immune-inflammatory response in the brain, further study on these parameters may provide newer insight into the treatment of stress conditions occurring after brain injury.

#### Intracranial pressure

Decompressive craniectomy reduces ICP and impairs the glymphatic inflow, which cranioplasty reversed in mice 75. In mice's early stage of ischemic stroke, PVS dilated because of vasoconstriction, doubling the glymphatic influx speed 76. The increase of the glymphatic inflow may cause the increased ICP induced by cerebral edema in the case of ischemic stroke; however, there is no direct conclusion characterizing the effect of increased ICP on the glymphatic influx. Another study reported that the glymphatic inflow was impaired after traumatic brain injury (TBI) 77. Although increased ICP is a severe complication of brain injury, this study did not attribute impaired glymphatic inflow to the increased ICP but instead to the impairment of AQP4 polarization 77. The decrease of ICP impairs the glymphatic inflow, and the increased ICP on it is still unclear. Some studies showed increased ICP after TBI generated meningeal lymphatic dysfunction, and the meningeal lymphatic drainage was impaired after stroke 16, 78, 79.

### Vasomotion

Some researchers believe that the efficiency provided by arterial pulsation should not exceed 15-25%, and vasomotion is a more efficient driving force 80-82. As another factor regulating the dynamic change of vascular diameter except for arterial pulsation, vasomotion is a spontaneous low-frequency (0.1-0.4 Hz) rhythmic cerebral vasoconstriction and dilation of the vascular smooth muscle cells motions 83. Functional hyperemia induced by visual stimulation increases the amplitude of vasomotion and promotes the clearance of fluorescent molecules in mice brains 82. APP / PS1 mice show impaired vasomotion and decreased clearance of fluorescent molecules during functional hyperemia 82. The dysfunction of the smooth muscle cells may be part of the possible causes of clearance dysfunction in the case of cerebral amyloid angiopathy 82. In addition, the inhibition of vasomotion during GA may be the reason for the decline of brain clearance function 74, 82. Vasomotion is not an isolation parameter and also intrinsically relates to other physiological parameters. According to the underlying principle of resting-state blood oxygen level-dependent signal in functional magnetic resonance imaging of rodents, the level of cerebral oxygen regulated by physiological parameters such as respiration and heart rate directly affects the movement of vascular smooth muscle 84.

### Aquaporin-4

**Figure 5:**
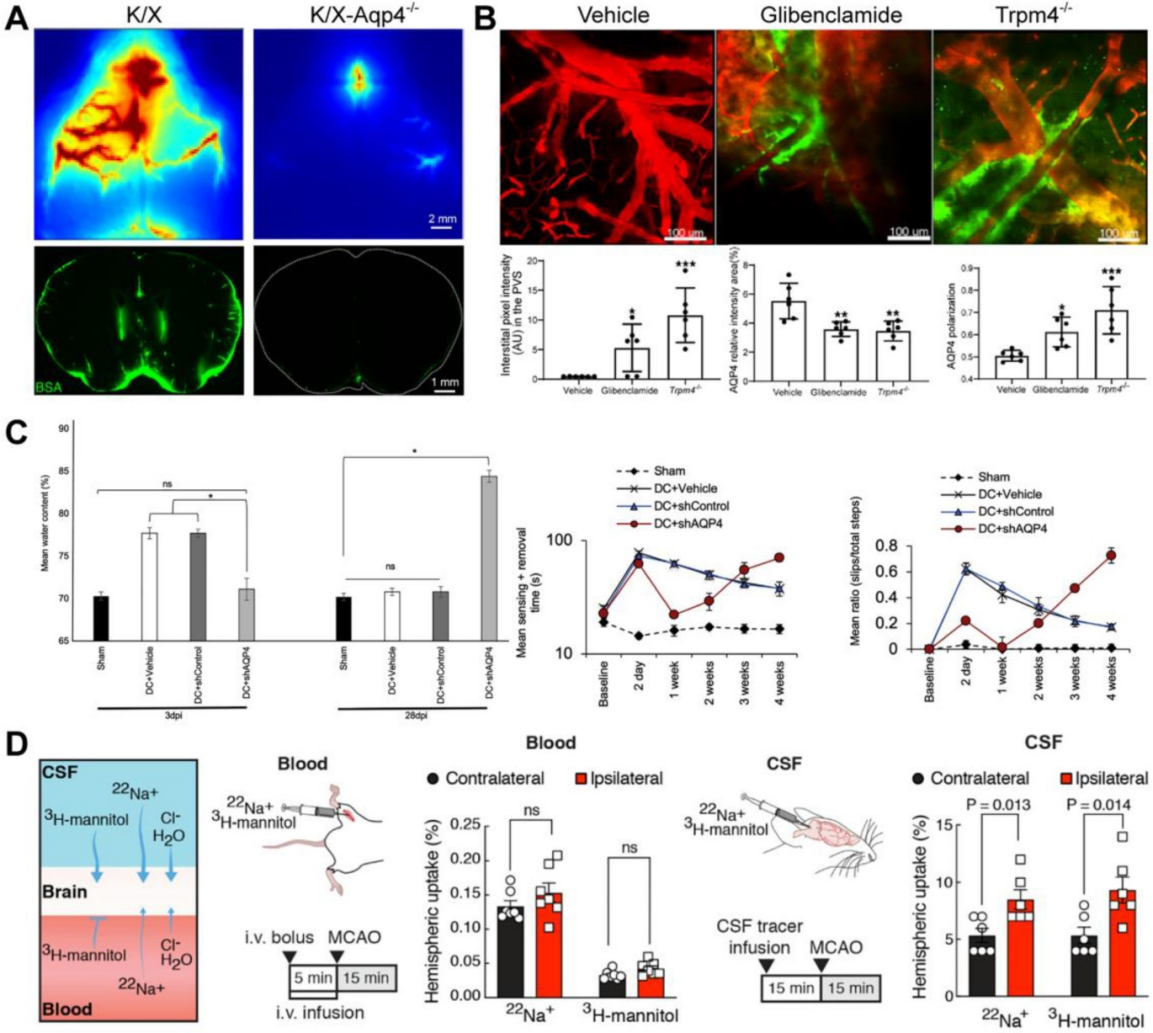
**Effects of AQP4 on different CNS diseases.** (A) The deletion of the AQP4 gene reduces CSF tracer influx. (B) Glibenclamide alleviates brain oedema, increases the polarization of AQP4, and reverses the impairment of GS function after SE. (C) AQP4 knockout reduces oedema and restores sensorimotor function on the 3-day post-SCI, while it increases oedema and deteriorates sensorimotor function on the 28-day post-SCI. (D) Labelling either the blood or CSF compartment shows that CSF is the source of the edema fluid. Adapted with permission from Ref. 90 for A), copyright 2018 American Society for Clinical Investigation; Ref. 93 for B), copyright 2021 American Society for Clinical Investigation; Ref. 97 for C), copyright 2020 Cell Press; and Ref. 76 for D, copyright 2020 American Association for the Advancement of Science.

Mammalian aquaporins, transmembrane proteins that promote the bidirectional transport of water across the cell membrane, are the primary water homeostasis regulators and participate in molecular transport and membrane protein expression, cell adhesion, and cell volume regulation 85. A subgroup of aquaporins water channels (e.g., aquaglyceroporin) also facilitate transmembrane diffusion of small, polar solutes except for water molecules 86, 87. AQP4, the most expressed aquaporins in CNS, is enriched on astrocytes end-feet and plays a vital role in the GS 38. According to the astrocyte-neuron lactate shuttle hypothesis, neural activity is related to brain lactate production to meet the higher energy requirements 2, 88. Lactate flow out of the brain through AQP4-dependent glymphatic clearance in the CSF mixture during rest 89. AQP4-null mice show markedly reduced CSF tracer influx into the PVS and clearance of metabolites in the ISS 3, 90 (Figure 5A). Notably, the perivascular polarization of AQP4 may play a more critical role. Snta1 knockout mice with regular expression and loss of polarization of AQP4 have the same inhibitory effect on glymphatic flow as AQP4 knockout mice 91. As described in 2.1.4, the circadian polarization change of AQP4 rather than the expression is related to the circadian change of GS function 17. The polar expression of AQP4 increases after voluntary exercise, which promotes the clearance of amyloid-β in mice 92. In some CNS diseases, glymphatic flow inhibition is also related to AQP4 depolarization. Inhibition of brain edema increases the polarization of AQP4 and reverses the impairment of GS function after status epilepticus (SE) 93 (Figure 5B). After TBI, the expression of AQP4 is delocalized, which impairs the glymphatic flow and increases the deposition of tau protein 77. The loss of AQP4 polarization and the impairment of the GS function still co-occur after 28 days of brain injury 77. AQP4 deficiency also aggravates the pathology of neurodegenerative diseases 94. Idiopathic normal pressure hydrocephalus, a subtype of dementia, is characterized by the loss of AQP4 polarization and the impairment of the glymphatic inflow, similar to those observed in AD mice 95, 96.

AQP4 also plays a vital role during brain edema. CNS edema is associated with increases in total AQP4 expression and AQP4 subcellular translocation after TBI, spinal cord injury (SCI), and stroke 97, 98. Trifluoperazine alleviates post-injured edema by inhibiting the expression and polarization of AQP4 after TBI, SCI, and ischemic stroke 97, 98. Importantly, this effect is achieved by targeting the calmodulin-mediated subcellular polarization of AQP4 97. The perivascular polarized expression of AQP4 plays a negative role by driving the cytotoxic edema caused by water influx in the early post-injury stage. Still, it also plays a positive role in promoting the clearance of vascular edema later 97. In a rat SCI model, AQP4 knockout reduced edema and restored sensorimotor function in the early stage but increased oedema and deteriorated sensorimotor function in the late stage 97 (Figure 5C).

Is there any intrinsic relationship between brain edema and GS transport? The source of water for brain edema is also a controversial issue. A recent study showed that CSF flowed into the ischemic area through PVS (the same inflow pathway as the GS), driving acute tissue swelling (Figure 5D) 76. Does the bulk influx of CSF into PVS synchronously lead to an enhancement of GS function and increase of solute clearance of ISS during acute ischemic (within 30 minutes as the study focused on 76)? Brain edema is long-term and complex pathophysiology, including cytotoxic edema, ionic edema, and subsequent vasogenic edema with the breakdown of the BBB 97. The GS function is impaired rather than enhanced, accompanied by pathological brain edema in the early stage (1-7 days) after TBI, ischemic stroke, and epilepsy in wild mice 77, 93, 99. The deletion or depolarization of AQP4 is adverse for vascular edema clearance and GS function recovery in brain injury. Obviously, brain edema and glymphatic transports are two different pathophysiological processes. Who led to the later differentiated outcome when they shared the same initial pathway (the glymphatic inflow)? In addition to the increase of ICP and the destruction of BBB caused by brain edema, the regional differential expression, inconsistent expression and polarization, and the repolarization of subcellular of AQP4 after brain injury also needs to be further characterized. AQP4 expression is strongly downregulated in the infarct and overexpressed in the penumbra after acute ischemic stroke in mice 100. Both patients with temporal lobe epilepsy associated with unilateral hippocampal sclerosis and mice in the early days after SE showed up-regulated AQP4 expression and decreased AQP4 polarization 93, 101. Other studies have shown that the increased AQP4 membrane localization in primary human astrocytes was not accompanied by a change in AQP4 protein expression levels 102, 103.

AQP4 inhibitors may benefit from the reversible blockade of AQP4 within one week after brain injury 97. Since AQP4 gene-deficient mice showed a coincident decrease between brain edema and GS clearance function accompanied by decreased AQP4 expression and polarization 102, 104, modulators of AQP4 might also be potentially effective drugs that reversibly inhibit cytotoxic edema in the early stage of brain injury but, after that, recover vascular edema clearance and GS function by AQP4 relocalization. However, despite intense efforts over many years, many highly hoped pore-blockers of AQP4 previously developed were later challenged because the inhibitory effects of most of these molecules were not repeatable in other analyses 38, 39. TGN-020 has originally designated an AQP4 inhibitor based on data from the Xenopus laevis oocyte swelling assay. However, when tested in cell-based assays, it lacks AQP4 inhibitory function 105, 106. Targeting AQP4 subcellular relocalization (a dynamic process independent of changes in AQP4 expression 39) may be an alternative strategy. Trifluoperazine eliminates brain edema in the CNS by inhibiting calmodulin, which drives AQP4 cell-surface localization by binding to the carboxyl terminus of AQP4 97. Glibenclamide alleviates pathological brain edema after SE by inhibiting the SUR1-TRMP4 channel complex on the astrocyte membrane and recovers GS function 93. Technical innovations will solve the attrition challenges in drug screening and confounding factors in water permeation analysis, such as calcein fluorescence queuing, human microvessel-on-a-chip platforms, high-throughput screening, and computer-aided drug design 107-110. The latter two have recently been applied to discover novel drugs for neurodegenerative diseases. They are likely to provide a novel insight that can help new treatments' findings targeting AQP4 in the future.

## Modulation of lymphatic efflux

### CSF production

CSF is mainly produced by the choroid plexus of the ventricular system and other tissues (such as the BBB) 111. Various physiological factors and molecules affect the production rate of CSF, and the production of CSF increases in some pathological conditions such as stroke and meningitis 112, 113. As the leading participant in the glymphatic inflow, it is vital to understand whether the CSF production modulates the GS.

The direct measurement of CSF production in mice by blocking the aqueduct of Sylvius indicated that isoflurane caused a higher rate of CSF production than that of the K/X cocktail 113. However, the increased CSF production does not indicate the increased glymphatic inflow since previous studies have shown that the K/X cocktail resulted in more glymphatic influx than isoflurane 26, 113. CSF production in female mice is higher than in male mice, but there is no sex difference in the glymphatic influx among the healthy young, middle-aged, or old mice 113, 114. These results expounded no association between the glymphatic inflow and CSF production. Still, this conclusion needs to be further studied since isoflurane can also cause changes of many factors (physiological parameters, fluid dynamics and vascular compliance), which may offset the benefits of increased CSF production.

In contrast, although there is no direct research evidence, other studies have demonstrated that the increased CSF production was consistent with the increased glymphatic influx. The production of CSF increases during the mid-rest phase and the treatment with non-selective β-adrenergic receptor antagonists in mice 113, 115, 116. Coincidently, the glymphatic influx increases during these conditions 14, 17. Both CSF production and glymphatic influx are reduced in elderly and AD mice 18, 113, 117, 118. The loss of CSF induced by cisternostomy and the reduction of CSF production treated with acetazolamide decrease the clearance of fluorescent protein and TBI injury markers in mice 89, 119.

We speculate that the regulation of CSF production under physiological conditions may be intrinsically related to the modulation of sleep, ANS, physiological parameters, and cerebrovascular, all of which ensure a stable and adequate brain clearance. Changes in CSF production during pathological conditions may be related to brain clearance disorder's compensatory or decompensated mechanism.

### Pathway of lymphatic efflux

CSF in the ventricles converges into the fourth ventricle through the interventricular foramen, the third ventricle, the midbrain aqueduct, and eventually flows into the cisterna magna circulate in SAS. CSF in the fourth ventricle can also flow into the central canal of the spinal cord. The drainage of CSF into the spinal cord is far less than that into the cranial cavity under physiological conditions 120, 121. Initial studies that ignored the effect of injection volume and rate on ICP suggested that the primary drainage route of CSF involved its return to the sagittal sinus via the arachnoid granules 122. Actually, no CSF flow into the superficial cerebral venous system through the arachnoid granules in physiological ICP conditions 6. There are three widely accepted pathways for CSF efflux from the cranial cavity (Figure 1): (1) along the olfactory nerve through the cribriform plate to the nasal mucosa, efflux into the superficial and deep cervical lymph nodes (sCLNs, dCLNs) (Figure 1A); (2) through the peripheral pathways of the trigeminal nerve, glossopharyngeal nerve, vagus nerve, and other crucial cranial nerves, drain into the sCLNs and dCLNs eventually (Figure 1B, blue dashed box); and (3) drainage through the MLVs into the dCLNs 4, 6, 123 (Figure 1C). Notably, recent studies have characterized specialized lymphatic structures on the dura mater passing through the vicinity of the cribriform plate and cranial foramen of the cranial base nerve 11, 124 (Figure 1A-B). The cranial nerves overlap anatomically with the base MLVs. These lymphatic vessels may be independent of cerebral nerves, and the excision of nerves will not affect MLVs' integrity and drainage efficiency 11 (Figure 1B, red dashed box).

The novel pathway, GS, elaborates that some CSF flow into the brain parenchyma before efflux out of the skull cavity 125 (Figure 1D). BBB, composed of tightly connected endothelial cells, astrocytes, and pericytes, provides a robust physical barrier for CNS and prevents foreign molecules' interference on neuronal activity and signal transduction 126. On the side, this barrier makes it impossible for the brain' metabolites to be discharged in the same way as the peripheral microcirculation. The GS provides a practical clearance pathway to the brain in a state of continuous high metabolic activity, especially those far away from BBB. It is certain that not all CSF have the opportunity to flow into the brain, and most of it directly outflows into the extracranial lymphatic vessels during awake 9, 71. Similar to the peripheral lymphatic system, the CSF transport rate is higher in the awake state than during sleep, and the rapid CSF outflow leads to a decreased glymphatic influx 71. According to the principle of mass conservation, the increased CSF efflux will inevitably lead to a reduced glymphatic influx in homeostatic conditions.

In addition, these traditional and novel drainage pathways also have some outstanding open scientific issues that need to be further characterized. For example, is there an essential connection between the conventional drainage path of the perineural space and the MLVs located around these nerves? The exact contribution of these outflow pathways to CSF drainage remains clarified. What anatomical pathway does the ISF pass into the dorsal and base MLVs?

### Meningeal lymphatic vessels

Although the mechanism of how CSF is transported to MLVs is unclear, many studies showed that CSF primary outflowed cranial cavity through MLVs 6, 10. Some studies demonstrated that MLVs also regulated the GS. In elderly mice, the density of the dorsal MLVs is reduced, and abnormal branching hyperplasia of MLVs at the base skull is noted, resulting in decreased CSF outflow to the dCLNs and reduced glymphatic inflow into the brain 6, 11, 127, 128. After blocking the MLVs outflow pathway of CSF with ligation of dCLNs, photochemical ablation of the MLVs or MLVs developmental defects, both glymphatic influx and brain solute clearance are decreased in mice 4, 10, 127-130. Overexpression of vascular endothelial growth factor C promotes the proliferation and remodeling of MLVs, which effectively reverses the reduced glymphatic influx caused by the aging-related degeneration of MLVs 127, 131.

**Figure 6:**
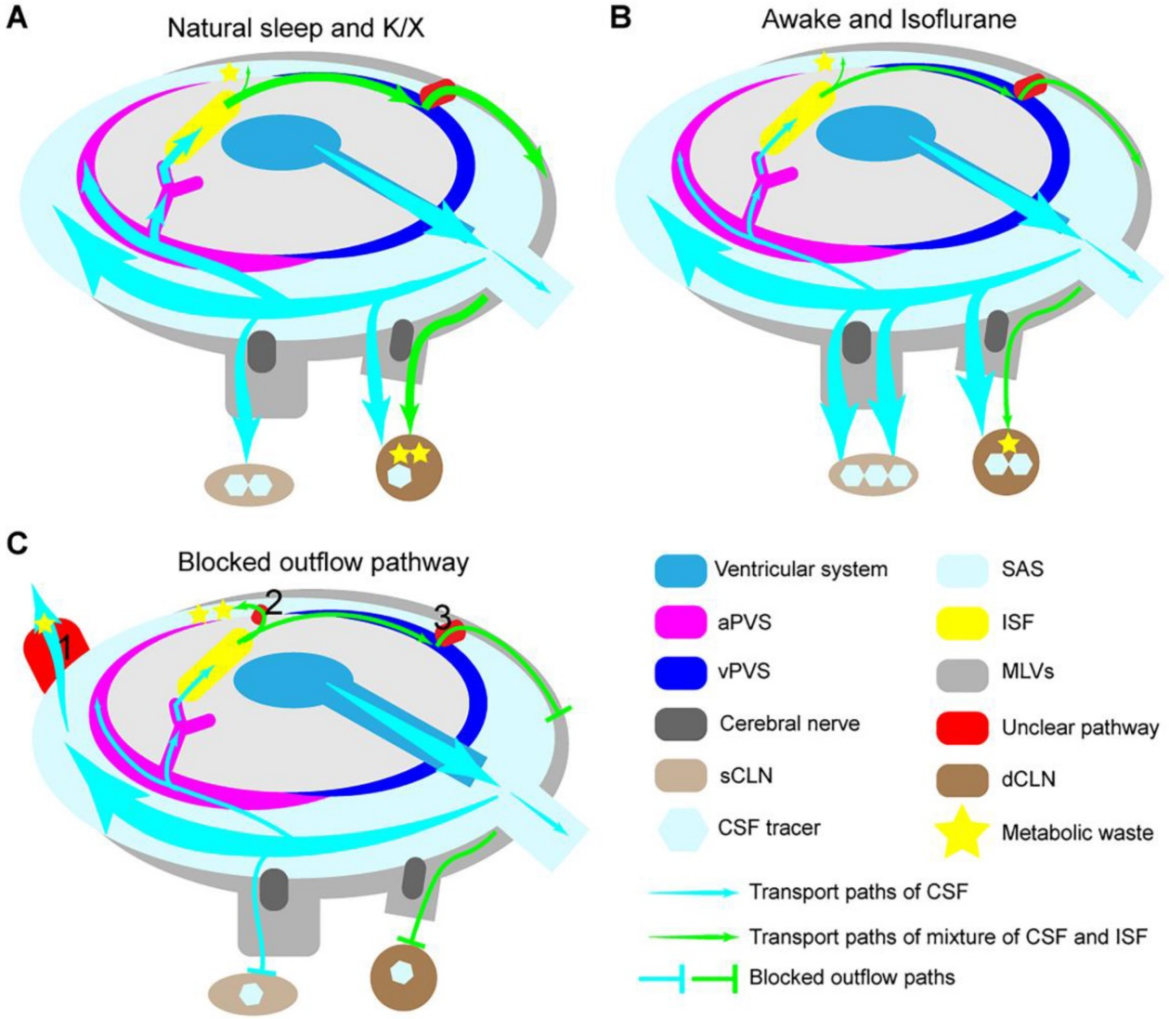
** The modulation of lymphatic fluid efflux in the cranial cavity.** (A) During natural sleep or K/X anesthesia, more CSF flows into the brain and efficiently removes accumulated metabolic waste through the GS pathway. (B) During awake or isoflurane anesthesia, the rapid outflow of CSF reduces the glymphatic inflow and brain waste clearance. (C) Blocking the outflow pathways impairs the glymphatic influx and reduces lymph drainage but does not increase ICP and interstitial hydraulic pressure. Some supplementary pathways may be involved in the shunt of CSF (red box 1) and ISF (red box 2) under some pathological conditions.

The degeneration of MLVs in aging mice and the blockade of the CSF outflow pathway does not increase brain water content and ISF pressure 4. This conclusion indicates that the MLVs' status as the primary CSF exclusion pathway will be challenged when dysfunctional. Other routes must diverge some CSF (Figure 6C, red box 1). When blocking the MLVs drainage pathway, the elimination of metabolic waste in the brain is not entirely blocked, which means the presence of other routes for the elimination of metabolic waste 132 (Figure 6C, red box 2). Small molecules injected into the ventricle or cisterna magna symmetrically drain into the bilateral dCLNs and sCLNs through the cribriform plate 4 (Figure 6A). Moreover, the tracers injected into the brain parenchyma primarily flow into the ipsilateral dCLNs, twice the contralateral, and less than 30% are circulated into the cisterna magna 4, 133 (Figure 6C). T cells injected through cistern magna will drain into the dCLNs and sCLNs, while those injected into the brain parenchyma could not be emptied into the sCLNs through the cribriform plate 10. Blocking the main outflow path of CSF reduces glymphatic influx synchronously, while the rapid outflow of CSF is inversely proportional to the glymphatic inflow; these are two contradictory conclusions (Figure 6B-C). Therefore, we propose the following hypothesis that may explain these results: the outflow pathway of CSF in the SAS may differ from that in the brain. Other complementary courses might become active when pathologically blocked outflow pathways (Figure 6C). CSF in the SAS may flow out of the cranial cavity symmetrically from the widespread outflow pathway, and the mixed fluid in the brain tends to drain into the ipsilateral side through a specific path. Further study on the anatomical track of CSF passing into the MLVs may help us understand these unclear ways.

Moreover, the proliferation and remodeling of MLVs have both sides in CNS disorders. Structural and functional disorders of MLVs will impair the brain clearance system and reduce the immune-inflammatory response, which may be a potential therapeutic target for CNS autoimmune response such as multiple sclerosis 124. In contrast, promoting the proliferation and remodeling of MLVs may be an effective treatment direction for intracranial tumors and neurodegeneration 127, 131, 134, 135.

## Conclusion

In this review, we systematically review the physiological regulatory mechanisms of the lymphatic transport in the CNS based on the two-process model of sleep regulation. It is now clear that no single pathological feature can explain this complex modulatory process in isolation. These mechanisms are related and interact upstream and downstream (Figure 2). Sleep is regulated by homeostasis (process S) and circadian rhythm (process C). The two-process and related factors (SWA, ESF, sleep deprivation, etc.) are all involved in the modulation of the glymphatic inflow. The mechanism of the differential regulatory effects of different anesthesia and sleep in the upstream is unclear, and there may be an interpretable mechanism in the downstream. During natural sleep, the reduced sympathetic tone and increased parasympathetic tone inhibit noradrenergic receptors. Then, following a decrease in respiratory rate and heart rate. The reduction of respiratory rate leads to increased CO_2_ partial pressure; after that, through internal chemical regulation mechanisms, the cerebrovascular dilation and vascular compliance increases, along with the lowering of heart rate, which eventually increases the arterial pulsation amplitude 136.

Unlike physiological regulation, pathological changes are often compensatory or decompensated physiological reactions occurring after disease onset. Like inflammation, which has both protective and adverse effects, we need to investigate the comprehensive impact of these physiological regulations on different diseases and stages after pathological CNS disorders and further identify the regulatory strategies that can be the most beneficial. These physiological parameters will also change during pathological stress after brain injury in different periods throughout the disease course. Many clinicians have been developing individualized and accurate therapeutic schemes by adjusting these physiological parameters to reduce secondary brain injury, and these therapeutics are also controversial. For example, which is a better therapy after severe TBI, the Lund concept, or the Brain Trauma Foundation guidelines 137? Is preventive hypothermia beneficial after TBI 138? As sleep has a restorative physiological function, the independent or comprehensive study of these modulatory mechanisms may identify critical targets for the recovery from CNS disorders.

Under physiological conditions, the rapid outflow of CSF reduces the glymphatic influx. Pathologically blocking MLVs does not increase cerebral water content and glymphatic influx. We speculate that the outflow pathway of CSF in the SAS may differ from that in the brain; other complementary routes may be hidden. Further studies that characterize the exact anatomical structure of these outflow pathways will help us find new therapeutic strategies that effectively regulate these pathways to counteract the brain damage caused by the changes of outflow pathways in pathological conditions.

The lymphatic transport may be involved in most pathophysiological processes in the CNS, so these directions are far from enough. We can restudy any CNS disease by targeting the specialized lymphatic transport system with new information and insight.

## Abbreviations

AD: Alzheimer's disease
ANS: autonomic nervous system
aPVS: artery paravascular space
AQP4: astrocyte aquaporin-4
BBB: blood-brain barrier
CBF: cerebral blood flow
CNS: central nervous system
CSF: cerebrospinal fluid
dCLNs: deep cervical lymph nodes
EEG: electroencephalogram
ESF: Endogenous sleep factors
GA: General anaesthesia
GABA: γ-aminobutyric acid
GS: glymphatic system
ICP: intracranial pressure
IPSCs: inhibiting postsynaptic currents
ISF: interstitial fluid
ISS: interstitial system
K/X: ketamine/xylazine
MLVs: meningeal lymphatic vessels
NREM: non-rapid eye movement
SAS: subarachnoid spaces
sCLNs: superficial cervical lymph nodes
SCI: spinal cord injury
SE: status epilepticus
SWA: slow-wave activity
TBI: traumatic brain injury
vPVS: venous paravascular space
